# The effect of functional splinting on mild dysplastic hips after walking onset

**DOI:** 10.1186/1471-2431-5-17

**Published:** 2005-06-15

**Authors:** Henning Windhagen, Fritz Thorey, Heinrich Kronewid, Thomas Pressel, Dieter Herold, Christina Stukenborg-Colsman

**Affiliations:** 1 Department of Orthopaedic Surgery, Hannover Medical School, Annastift, Anna-von-Borries-Str.1, 30625 Hannover, Germany; 2 Trauma Surgery, Städtisches Krankenhaus Hildesheim, Weinberg 1, 31134 Hildesheim, Germany; 3 Department of Pediatric Orthopaedic Surgery, Clinic 1, Annastift, Anna-von-Borries-Str.1, 30625 Hannover, Germany

## Abstract

**Background:**

For treatment of Graf class IIb dysplastic hips at walking onset a treatment concept with abduction splints allowing patterns as walking and crawling under constant abduction control was investigated. However, as the splint still incapacitates child movements the research question remains whether the physiologically progressing maturation of hips can be significantly altered using such abduction splints for walking children.

**Methods:**

Of 106 children showing late hip dysplasia, 68 children treated with the Hoffman-Daimler (HD-splint) abduction splint were compared with 38 children with neglect of the abduction treatment in this retrospective study. Radiographic analyses were performed measuring the development of the age dependent acetabular angle.

**Results:**

The regression analysis for splint treatment showed a significant linear regression for both splint treatment and no splint treatment group (r^2 ^= 0,31 respectively r^2 ^= 0,33). No statistical difference between both treatment groups was apparent.

**Conclusion:**

Considering the characteristics of this study, there seems to be no strong rationale supporting the use of an abduction device in growing children. As no significant difference between treatment groups is apparent, a future controlled prospective study on splinting effects can be considered ethically allowed.

## Background

Developmental dysplasia of the hip is a gradually progressing disorder reflecting anatomically different situations reaching from mild subluxation of the femoral head to full luxation of the hip. The disorder is caused by malformations of anatomic structures that have developed during the embryologic period. The pathology of developmental dislocation of the hip is associated with a loose hyperelastic capsule, elongated ligamentum teres and slight eversion of the hypertrophied acetabular rim. While the femoral head is normal in shape, excessive femoral and acetabular antetorsion may be present causing anatomic instability of the hip joint [1-5].

Subluxation and luxation are conditions that always lead to symptomatic degenerative hip disease [5-7]. Depending on the severity of dysplasia pain onset is observed already in the second decade for severely subluxed hips while minor subluxation leads to pain starting in the fifth or sixth decades. Considering this time course of disease early treatment regimens for developmental dysplasia of the hip were recommended.

While dislocated hips after birth present clinical features as the Ortolani's and Barlow's sign [8], subluxated hips present significant changes in the sonographic morphology of the hip. Therefore, especially in Europe, ultrasound is considered to play a pivotal role in the early diagnosis of developmental hip diseases [9,10]. Especially in babies with risk factors associated with developmental dysplasia a careful examination is needed [11]. General screening concepts remain controversial due to added costs [12,13]. The treatment of dysplastic hips depends on the degree of subluxation. Based on the sonographic appearance the Graf classification has gained wide acceptance [14-18]. While class I hips need no follow up and treatment, class II hips form a group in which the degree of abnormality and the need for treatment are less clear and remain controversial. While some authors treat class II hips showing instability [19], others report about spontaneous recovery [20]. For treatment purposes authors introduced abduction devices such as harnesses providing abduction and flexion [20-22]

Graf Class II b hips are defined as hips of babies older than three month, exhibiting an alpha angle of 50–59 degrees. Radiographs show an acteabular angle of more than 30 degrees. The morphology shows a stable, but deficient bony shape of the acetabulum and femur and a broadened cartilage roof. Class IIb hips show a deficit of bony maturation and therefore need treatment options. Usually, the use of abduction devices is expanded until walking onset at approximately age 8 month. With the increased mobility of the baby, an abduction and flexion harness becomes an increasing handicap.

For treatment of Graf class IIb hips at age of walking onset an abduction splint with ball and socket joints was introduced, allowing patterns as walking and crawling under constant abduction control. However, the splint still incapacitates child movements and is generally not liked by parents and custodians. Thus, an estimated number of untreated cases can be considered leading to the research question, whether the physiologically progressing maturation of hips can be significantly improved using such abduction splints for walking children.

## Methods

**Figure 1:**
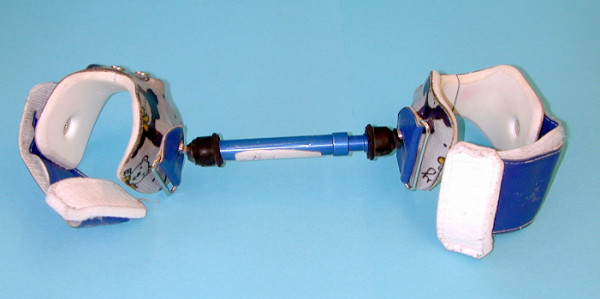
**Hoffman-Daimler abduction splint. **The Hoffman-Daimler splint consists of a individually shaped leg sleeve and a variable bar connected by ball and socket joints.

Between1998 and 2004 106 children were treated with the Hoffman-Daimler (HD-splint) abduction apparatus (Fig. 1) and included in this retrospective study. The age at diagnostics was 6–18 month. Indications of treatment were based on sonography for children under 8 month and plain a.-p. radiographs over age 8 month. To allow a combination of ultrasound and radiographic measurements, the dysplasia classification of Tönnis and Brunken [1] was used.

A treatment algorithm based on the Graf Classification was introduced in the Department of Orthopaedic Surgery in 1989. The use of HD-splints in Class IIb hips was fully established prior to this retrospective analysis. From 1998 68 children were treated using the HD-splint, while no splint was used in 38 children. All children were monitored using radiographic follow ups of the hips. Reasons for no splint treatment were solely based on parents or custodians declared intention. The decision, not to use a splint was argued with pain, incapacitation, poor splint fitting and continuous neglect.

**Figure 2:**
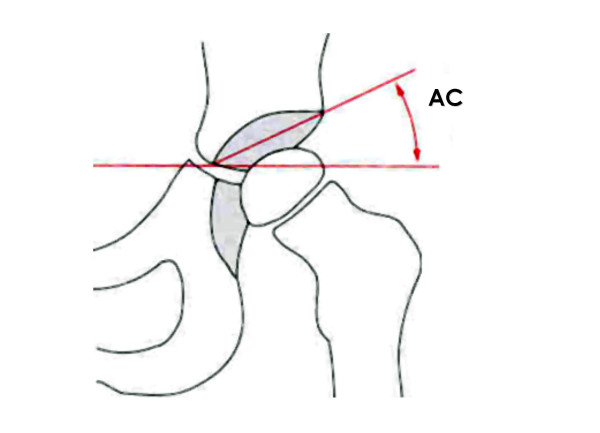
**Acetabular angle (AC – Hilgenreiner). **The AC- angle arises from a horizontal line trough the triangular cartilage at the right and left pelvis and a second line connecting the corner of the triangular cartilage and the lateral acetabular rim.

Radiographs were digitized using a radiographic scanner and analyzed using ImageJ -software (NIH, Bethesda, USA). The acetabular angle (AC) following Hilgenreiner was measured for all hips and assigned to the respective radiographic age. (Fig. 2).

Regression analyses were performed between age as dependent variable and AC-angle as independent variable. Differences in regressions between the no splint group and the abduction splint group were compared using an analysis of covariance.

## Results

### Classification

88% of all hips were graded mild dysplastic (1 standard deviation, [1]) following screening data of mid-European newborn hips, while 12% were graded severely dysplastic (2 standard deviations, [1]). Those hips with treatment start under 8 month were sonographically classified Graf IIb, those over 8 month were graded by x-ray anaylsis (AC-angle).

### Regression analysis

**Figure 3:**
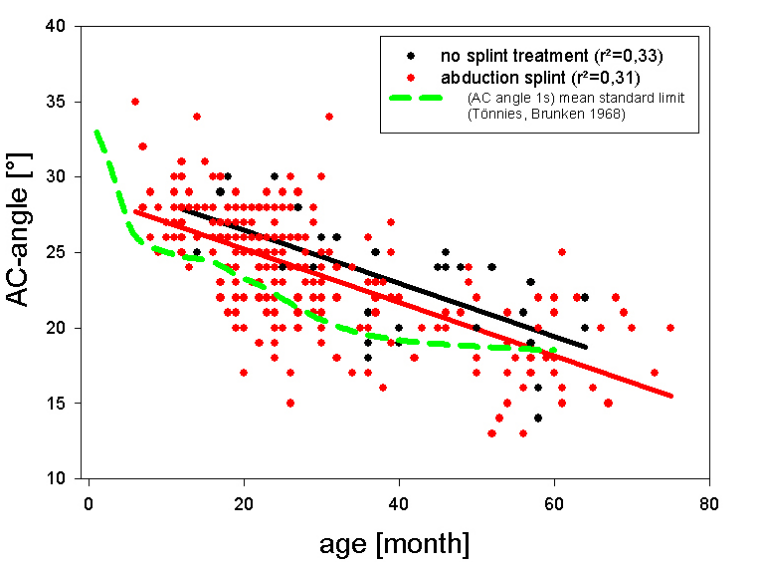
**Regression analysis of treatment groups. **Regressions of the splint and no splint groups are displayed with age as the dependent and AC-angle as the independent variable. For illustrative purposes the pathological limits for age dependent AC-angles from literature data [2] are displayed. Side- and gender-related differences were averaged. Regression equation and coefficient of determination (r^2^) are displayed.

The regression analysis for splint treatment showed a significant linear regression for splint treatment. (y = 29,9x-0,17, p = 0,03; r^2 ^= 0,31). Similarly, the regression analysis for the no splint treatment group showed a significant regression for splint treatment (y = 28,7x-0,176, p = 0,03; r^2 ^= 0,33). There was no difference between both treatment groups regarding regression quotients as analyzed with ANOVA. (Fig 3.)

Initial AC-angles in the splint treatment group were higher than in the no splint group (mean 29 ± 8 vs. 26 ± 6)

Over time both groups showed a decline of the acetabular angle (AC) from 26+-6 degrees at 12 month to 17 ± 5 degrees at 70 month.

## Discussion

The goal of this study was to assess the effect of an abduction splint during onset of walking age in children on the maturation of dysplastic hips. In a retrospective study on 106 children no difference was obtainable between a group of children treated with this abduction device and a group of children neglecting such devices. From the data of this study no rationales were found indicating a sufficient effect of this abduction device in the presented treatment context.

The concept of continuous abduction in growing children with mild dysplastic hip symptoms was introduced before [23,24]. As much as the effect of early abduction devices (harness etc.) is known, several authors further recommend the use of rocking horses and toys with abduction for late cases of hip dysplasia [25]. However, comparing abduction devices of young babies with the presented abduction splint one has to consider a major difference: Abduction splints in young babies always combine abduction with hip flexion. With walking onset the children's legs are abducted by approximately 60 degrees using the HD-splint, however no flexion is apparent. Thus, one reason for no visible effects of the HD-splints may be the missing flexion component.

Another reason for the analogy of results in both groups may be based on the group assortment and group size. The design of this study was purely retrospective and differences between both groups were developed by the behavior of children and parents. Furthermore the groups were not balanced regarding the initial magnitude of the AC-angles. With slightly higher initial AC-angles in the HD-splint group an influence of the splint in decreasing AC-angles could be underestimated in this analysis.

The reason, why babies reach walking onset with symptomatic hips despite detection and treatment may have several reasons: First, the diagnosis may be late and abduction therapy introduced several month after birth; second, initial errors in ultrasound screening may have underestimated the hip condition and led to neglect of sufficient therapy. As a third reason the insufficient development of hips initially graded class I has been described. In these patients the maturation of the acetabular roof does not progress sufficiently. Causes of this development remain unclear.

When interpreting the results of this study several shortcomings have to be addressed: first, the treatment group were different with 36 to 68 group size. Second, the radiographic follow ups for each patient were different, thus there is a tendency to overweight patients with multiple follow ups in this study. Third, the initial therapeutic decision for splint treatment happened during an age range form 6 to 18 months. Thus, the comparison of patients with different ages of treatment initiation may influence the validity of the presented data.

## Conclusion

The effect of an abduction splint during onset of walking in children on the maturation of dysplastic hips remains unclear. From data of this study there is no strong rationale supporting the use of an abduction device in growing children, however, the results have to be weighted according to the characteristics of the study design. On the other hand, this study gives strong support for the ethical validity of a controlled prospective study, as no siginifcant difference between treatment groups can be expected.

## Competing interests

The author(s) declare that they have no competing interests.

## Authors' contributions

HW and HK designed the study profile, participated in the patient selection, data assessment and drafted the manuscript. TP provided radiographic digitization and angle measurements. FT participated in mathematical analysis. DH participated in the design of the study. CS participated in its design and coordination and helped to draft the manuscript. All authors read and approved the final manuscript.

## Pre-publication history

The pre-publication history for this paper can be accessed here:


